# Data on spatiotemporal urban sprawl of Dire Dawa City, Eastern Ethiopia

**DOI:** 10.1016/j.dib.2017.04.008

**Published:** 2017-04-13

**Authors:** Chaltu Taffa, Teferi Mekonen, Messay Mulugeta, Bechaye Tesfaye

**Affiliations:** a Adama Science and Technology University, Ethiopia; b Kotebe Metropolitan University, Ethiopia; c Addis Ababa University, Ethiopia

**Keywords:** Urban sprawl, Land use, Land cover, Peri-urban, Dire Dawa, Ethiopia

## Abstract

The data presented in this paper shows the spatiotemporal expansion of Dire Dawa City (eastern Ethiopia) and the ensuing land use land cover changes in its peri-urban areas between 1985 and 2015. The data were generated from satellite images of Thematic Mapper (TM), Enhanced Thematic Mapper-Plus (ETM+) and OLI (Operational Land Image) with path/raw value of 166/053 by using Arc GIS 10.1 software. The precision of the images was verified by geolocation data collected from ground control points by using Geographic Positioning System (GPS) receiver. Four LULC classes (built up area, vegetation, barren land and farmland) with their respective spatiotemporal dimensions were clearly identified in the analysis. Built up area had shown an overall annual increment of 15.8% (82 ha per year) from 517 ha in 1985 to 2976 ha in 2015. Expansion took place in all directions but it was more pronounced along the main road towards other nearby towns, recently established business/service areas and the Industrial Park. Barren land, farmland and vegetation areas showed speedy decline over the years.

**Specifications Table**

**Table:** 

Subject area	Urban study, geography
More specific subject area	Land use land cover change, urban sprawl
Type of data	Table, figure and text file
How data was acquired	Data were extracted from TM, ETM+ and OLI images with path/row values 166/053 and firsthand data were acquired by using Google Earth and GPS-based ground survey technique.
Data format	Analyzed
Experimental factors	
Experimental features	The images were geo-referenced with World Geodetic System (WGS) 1984 datum and Universal Transverse Mercator (UTM) projection system zone 37 North. The images were classified based on visual interpretation and supervised classification using Arc GIS 10.1 software.
Data source location	Dire Dawa City (8°52′–8°56′N, 38°48′–38°52′E) Landsat, OLI, and Google Earth
Data accessibility	The data is with this article

**Value of the data**

• The data is helpful to Dire Dawa City administrators to speculate the extent of the spatiotemporal expansion of Dire Dawa and its potential impacts on the surround areas.
• The data provides information on the status of urban expansion towards rural peri-urban areas around Dire Dawa City.
• The data is vital to model urban expansion towards rural peri-urban areas surrounding Dire Dawa City to mitigate its adverse impacts on the livelihoods of the people inhabiting the area and the ecosystem.
• The data is useful to researchers, urban planners and experts working in the field.

## 1 Data

**Fig. 1:**
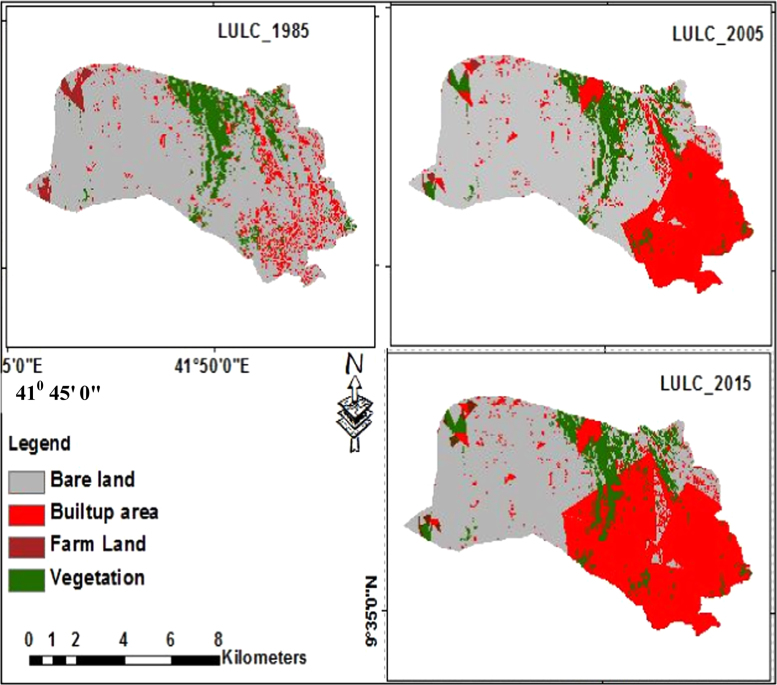
LULC map of Dire Dawa City in 1985, 2005 and 2015.

**Fig. 2:**
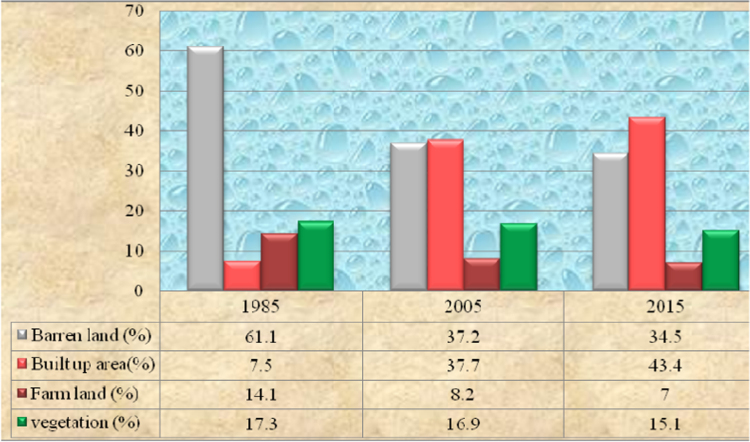
Chronological distribution of land use land cover area in percent in 1985, 2005 & 2015.

The data in this article provides information on the spatiotemporal expansion of Dire Dawa City (eastern Ethiopia) and the ensuing LULC changes in its peri-urban areas between 1985 and 2015. Fig. 1 illustrates pictorially the spatiotemporal extent of the LULC classes of the area in 1985, 2005 and 2016. Fig. 2 and Table 1 show that barren land covered 4195 ha, built up area 517 ha, farmland 967 ha and vegetation 1183 ha in 1985. The extent and rate of LULC change of the area are presented in Table 2, Table 3, Table 4. The change detection between 1985 and 2005 (Table 2) indicates that built up area increased from 517 ha in 1985 to 2585 ha in 2005. Barren land decreased from 4195 ha in 1985 to 2558 ha in 2005, farmland decreased from 967 ha in 1985 to 561 ha in 2005 and vegetation decreased from 1182 ha in 1985 to 1158 ha. Change detection between 2005 and 2015 (Table 3) shows that only built up area increased from 2585 ha in 2005 to 2976 ha in 2005; while all the rest LULC classes show declining change. Change detection for the entire study period (Table 4), shows that built up area increased from 517 ha in 1985 to 2976 ha in 2015 with annual rate of 82 ha while all the rest LULC classes show a declining change.

## 2 Experimental design, materials and methods

TM (1985), ETM+ (2005) and OLI (2015) dry season *landsat* images of 30 m spatial resolution and path/row values of 166/053 as well as GPS-based ground survey records were vital data sources for this data article. The images were geo-referenced with World Geodetic System (WGS) 1984 datum and Universal Transverse Mercator (UTM) projection system zone 37 North. The analysis comprised of layer stacking, radiometric correction, image enhancement, haze reduction, band combination and false color combination. Google earth maps of each year were used for GPS-based ground verification with a minimum of 20 spatially distributed ground control points in the area. Reconnaissance survey and researchers׳ experience of the study area were also vital. With this pre-assessment, four LULC classification schemes such as built up area, farmland, vegetation and barren land were identified considering the standard classes defined by the US Geological Survey as well as the study detail and objectives [1], [2]. Supervised classification technique was used for all images to identify the class features of the image. Moreover, post classification technique was applied to enhance the brightness of the classified images. Classification accuracy assessment was made at post classification stage to evaluate how well the classified images represented the real world. At the end, urban expansion change detection map of each year was produced. The extent and direction of the city׳s expansion were analyzed by calculating the corresponding areas with Arc GIS 10.1 software and related statistical formula [1].

## Figures and Tables

**Table 1:** LULC classes and comparison in 1985, 2005 and 2015.

Land use classes	1985		2005		2015	
	Area (ha)	%	Area (ha)	%	Area (ha)	%
Barren land	4195	61.1	2558	37.2	2370	34.5
Built up area	517	7.5	2585	37.7	2976	43.4
Farmland	967	14.1	561	8.2	478	7
Vegetation	1183	17.3	1158	16.9	1038	15.1
**Total area**	**6862**	**100**	**6862**	**100**	**6862**	**100**

**Table 2:** Spatiotemporal LULC changes between 1985 and 2005.

Land use land cover classes	1985		2005		Change		Annual rate of change ha y^−1^
	ha	%	ha	%	ha	%	
Barren land	4195	61.1	2558	37.2	−1637	−23.9	82
Built up area	517	7.5	2585	37.7	+2068	+30.1	103.4
Farmland	967	14.1	561	8.2	−406	−5.9	20.3
Vegetation	1183	17.3	1158	16.9	−25	−0.3	1.25
Total area	6862	100	6862	100	0.0	0.0	

**Table 3:** Spatiotemporal LULC changes between 2005 and 2015.

Land use land cover classes	2005		2015		Change		Annual rate of change ha y^−1^
	ha	%	ha	%	ha	%	
Barren land	2558	37.2	2370	34.5	−188	−2.7	18.8
Built up area	2585	37.7	2976	43.4	+391	+5.6	39.1
Farmland	561	8.2	478	7	−83	−1.2	8.3
Vegetation	1158	16.9	1038	15.1	−120	−1.7	12
Total area	6862	100	6862	100	0.0	0.0	

**Table 4:** Spatiotemporal LULC changes between 1985 and 2015.

Land use land cover classes	1985		2015		Change		Annual rate of change ha y^−1^
	ha	%	ha	%	ha	%	
Barren land	4195	61.1	2370	34.5	−1825	−26,6	60.8
Built up area	517	7.5	2976	43.4	+2459	+35.8	82
Farmland	967	14.1	478	7	−489	−7.1	16.3
Vegetation	1183	17.3	1038	15.1	−145	−2.1	4.8
Total area	6862	100	6862	100	0.0	0.0	00
